# Miscellanies

**Published:** 1843-04-01

**Authors:** 


### MISCELLANIES.

### SUMMARY OF THE WEEKLY TABLES OF THE MORTALITY FOR 1842:—METROPOLIS.

FROM these highly interesting and instructive Tables, we shall proceed to give some extracts.

The mortality in London during the past year amounted to 45,272, viz., 22,841 males, and 22,430 females, which, taking the population as it was in 1841, gives as the annual rate of mortality per cent. 2.381. From this it appears that the year has been, on the whole, healthy, the mortality being lower than in any of the previous years for which the number of deaths is given. The mean temperature has been high, viz., 52° 3′, the amount of rain in inches nearly 22, or considerably above the average. With regard to the influence of situation, we find, as in former years, the West districts to be the most healthy, next the North, then the Central, and finally the South and East districts, comprising Bermondsey, Southwark, Rotherhithe, Shoreditch, Bethnal Green, &c., in which the mortality amounted to nearly 2.5 per cent.

With regard to the influence of the seasons on the health, we find that the first quarter of the year, or that in which the mean temperature is the lowest, is most fatal, the number of deaths being 12,396; next to that, the last quarter, then the third quarter, and finally, as the most healthy, the second quarter, or that including the latter part of Spring and the beginning of Summer. From this we see that, while the very cold months are decidedly the most fatal, still the warmest months are far from being the most healthy, the fewest deaths occurring in that quarter in which the temperature most nearly approaches the yearly mean.

On examining more particularly the causes of death, we find in the first Table, consisting of epidemic, endemic, and contagious diseases, but little to notice; the greatest mortality occurred in the third and fourth quarters. Hooping-cough and croup proved most fatal in the early months; measles, scarlatina and typhus, in the latter months. November and December present the greatest number of deaths from erysipelas; diarrhœa, dysentery and cholera prevailed most in August and September. On comparing the number of deaths from these causes with the weekly average for 1838-9-40, we find a great diminution, the rate having been reduced from 204 to 148; this reduction taking place principally under the heads of small-pox, typhus and scarlatina, the weekly average for the former having fallen from 36 to rather less than 7; in the second, from 46 to about 22.6, and in the third, from 38 to 23.5. Diarrhœa, dysentery and cholera exhibit an increase.

In the second Table, consisting of the deaths from diseases of the nervous system, we see but little influence exerted by the temperature or season of the year. The greatest number of deaths under this section is referred to convulsions, next to hydrocephalus, then to apoplexy, paralysis and cephalitis. The rate of mortality exhibits a slight diminution compared with that of previous years, having fallen from 151 to 144.3, the decrease being principally referrible to the head of *convulsions*.

The third Table consists of the diseases of the respiratory organs. Over bronchitis, pleurisy and pneumonia, the cold quarters exert the most fatal influence; in consumption, however, the case is changed, the Summer months are here the most dangerous; the times at which the greatest number of deaths takes place are April and May, and October; or, the periods at which the greatest changes in the temperature usually take place. This Table shows a slight increase in the mortality of the year, as compared with previous ones, the weekly average having advanced from 266 to 269.2.

With regard to the mortality from affections of the circulatory system scarcely anything can be said, as 989 out of 1046 are comprised under the vague term "disease." The weekly average under this heading has increased from 18 to 20.

The next Table consists of diseases of the digestive organs. The greatest mortality occurs here in those months which are characterized by the highest degree of heat. The diseases which proved most fatal were gastro-enteritis, teething, and disease of liver. The mortality has increased this year from 60 to 65 per week.

The Tables of the deaths from diseases of the organs of generation, of locomotion, and of the integumentary system, present but little interest. The number who died in childbirth amounted to 321, from rheumatism, 119.

The diseases of uncertain seat include a great variety, such as inflammation, hæmorrhage, atrophy, debility, &c. Of these the greatest number of deaths, viz., 1750 are attributed to dropsy, 1148 to debility, and 870 appear as "sudden deaths." The ratio of the mortality has increased from 103 to nearly 110, the increase occurring principally under the heading of "debility."

To "old age" 3346 deaths are attributed, the greatest number occurring, as might have been expected, during the cold season. Intemperance, starvation, and violence account for 1267; and in 190 cases the cause is not specified.

In reviewing these Tables generally, we find that by far the greatest mortality is caused by diseases of the respiratory system, the number of deaths from these amounting to as much as 13,990, forming nearly one-third of the whole. Next in point of mortality come epidemic, endemic, and contagious deaths, accounting for 7696, or rather more than one-sixth; then diseases of the nervous system, number of deaths 7505, or about one-sixth; then diseases of uncertain seat, amounting to 5715; then diseases of the digestive organs, number of deaths 3396. To old age are attributed 3346; to intemperance, starvation and violence, 1267; diseases of the circulatory system, 1046; of the organs of generation, 445; of the urinary organs, 323; of the organs of locomotion, 280; and of the tegumentary system, 73.

Compared with the average of the years 1838-9-40, the mortality has decreased from about 48,150 to 45,272. The decrease taking place principally in the cases of epidemic, endemic, and contagious diseases, and also, though to a less extent, in diseases of the nervous system. Under most of the other headings, a slight increase has taken place.

In conclusion, we wish to express our satisfaction at the accurate and careful manner in which these Tables are drawn up, forming, as they do, a most valuable and interesting addition to our statistical knowledge of disease.

---

### The Indian Medical Boon.

For some years past, the Medical officers of the Indian Establishments have been petitioning the authorities at home for what is termed, "The Boon;" meaning pension for length of service, such as in 1837 was granted to the officers of the Indian Army. This boon, however, on some pretence or other was withheld, and probably would have continued to be withheld for a long term of years, but for the unceasing efforts of Mr. Martin, of the Bengal Medical Establishment, who, from the moment of his arrival in England, set to work to place the matters of the boon, as well as the condition of the Medical Service generally, in their proper lights before the authorities. This was no easy affair, as all who have attempted to work their way through the great public offices of this Metropolis well know. But Mr. Martin succeeded at last—he obtained the following scale of pensions, for officers who previously, and whatever their length of service, possessed but £191. 12s. 6d. per annum:—

| | | | | | | | | |
|---|---|---|---|---|---|---|---|---|
| After | 28 | years' service | (3 years' | Furlough | included) | £300. | a year. |
| „ | 32 | „ | „ | „ | „ | „ | 365. | „ |
| „ | 35 | „ | „ | „ | „ | „ | 500. | „ |
| „ | 38 | „ | „ | „ | „ | „ | 700. | „ |

Now, as compared to the Military boon, it is certain that, in respect to the time of service which entitles to pension, the Medical officers stand at disadvantage, especially when we consider that these last enter the service at a more advanced period of life. The omission of increased pension also, for Medical officers of 21 years' service, seems unjust and impolitic; yet, these are points that, we are assured, a little time and a proper representation will cause to be rectified, so as to place the two services on a footing of equality.

That "the boon" should appear to many to fall short of their just expectation, we can readily imagine; but still *it is a boon,* and will be felt by many to be so. The older surgeon will readily understand in this expensive country, the difference between £191. 12s. 6d. and £700. per annum. But that this grant should be considered in the strange light which is exhibited in the letters of some of our Indian correspondents, does somewhat surprise us. We should be sorry to characterize, in terms they deserve, the lucubrations of some of our friends, and still more sorry should we be to publish their letters, for we are certain that, such publication would be the very severest punishment we could inflict on their authors. To take one sample out of many:—a gentleman writes us to request that we publish an article of our own, that should have the effect of keeping the promotion in Bengal low, retaining that of Bombay high!—of lowering the price of provisions in Bombay!—of lowering the rate of mortality at a certain station there!—of reducing the rate of wages for domestics; and he concludes by telling us that, the Court of Directors of the East India Company "insultingly call" the recent increase of pension "a boon!!" We are sure that a little information and some little reflection will satisfy any one of our brethren in India that, no credit or profit can ever accrue from representations of this sort.

What they have really to do is, first, to inform themselves as to what ought to be done to render their departments efficient; and, secondly, to unite in the endeavour to bring the thing about. The Indian Medical Establishments are full of able men. Let these take the direction of affairs on the spot, and matters will soon be concluded to their satisfaction at home.

But there are some points that ought to be insisted upon in their petitions, and which we beg to submit for their consideration:

1st. That whilst the Military officer may retire in his 24th year of service, and aged 40, on a pension of £292., the Medical officer of 24 years' service, and aged 46, has but a pension of £191.; and whilst a Military officer of 28 years' service may retire on Lieutenant-Colonel's pension, a Medical officer is obliged to serve one year longer for the same pension.

2nd. That, to bring the two services to something like a par, as to retiring pensions, the following scale would seem an equitable one for the Medical department:—

| Years of Service. | Pensions. | Age of Retirement. |
|---|---|---|
| 17 years .. .. | £191. 12s. .. .. | 39 years of age. |
| 21 „ .. .. | 290. „ .. .. | 43 „ |
| 25 „ .. .. | 365. „ .. .. | 47 „ |
| 29 „ .. .. | 450. „ .. .. | 51 „ |
| 32 „ .. .. | 500. „ .. .. | 54 „ |
| 35 „ .. .. | 700. „ .. .. | 57 „ |

3rd. That, by the existing scale of retiring pensions for the two services, there is a difference of three years in the lowest scale, in favour of the Military officer, and in two others of the scales there exists a difference of seven years respectively in favour of the Military officer; and this, although he enters the service six years earlier in life, and receives pay in India during the whole of the time that the Medical officer is undergoing at home a laborious and expensive course of preparation for his responsible duties—duties on the right performance of which the very existence of modern armies mainly depends.

4th. That the rank, without the pay, of Major, conferred on Medical officers of 30 years' standing, is a positive injury. Increased rank is no where so sure to entail increase of expenditure as in India; and, all things considered, it is hoped the circumstance has only to be brought to the notice of authority to secure for the Medical officer the rank and pay of Major at a period of life somewhat corresponding, and judged by the average ratio of promotion in the Army.

These and other points, well known to our brethren in the East, should be briefly and emphatically insisted upon. Hitherto the Indian petitions and memorials have been very much wanting in that compactness so necessary to secure attention here; and, it is owing to the brevity of Mr. Martin's representations, which he urged forward by every influence he could command, as well as by great personal exertions, that he has obtained a hearing from the authorities; but it is lamentable to think that, whilst Mr. Martin was struggling with difficulties at home for the good of his brethren in India, and that in a manner which those alone can appreciate who have had to carry points with persons in authority, or through the great public offices of this metropolis, the old men, his official superiors in Bengal—the three dull-brained constituted heads of the Medical Department of the Army—that inert mass of obstructiveness—the Board—were busying themselves in the miserable manner here described:—*(Vide Lancet, March 11th.)*

"INSUBORDINATION AT THE MEDICAL BOARD."

"There is a somewhat novel state of things, regarding the members of the Medical Board, at present under the consideration of the higher authorities, and which, immediately relating to the question of Military uniform, involves the higher one of Military authority. We shall relate one of the several accounts (not substantially varying) which we believe to be the most correct. Not very long since, Mr. Sawers, the senior member, considered of a sudden that, as there was a uniform for the Medical Staff, that uniform should be worn at all meetings of the Board, and he mentioned this desire to the other, or junior members, Doctors Campbell and Smith, and said, at the end of a fortnight (allowing that time for the uniforms to be prepared) they should appear accordingly. They considering this as a proposition rather than as an order, voted against it, and intimated to Mr. Sawers that his motion was negatived by the majority.

"He made no remark whatever upon this result, and such meetings as next ensued were attended in the old way—plain cloth coat, or white jacket, according to the 'warm feelings' of the respective members—until the first meeting occurred after the expiration of the fortnight's law, when, on Dr. Campbell's entering the office in a white jacket, Mr. Sawers, who was himself in undress uniform, ordered him to go home, and consider himself in arrest for disobedience of orders.

"Home he went accordingly, and there he has remained in arrest ever since, and charges have been sent in against him by Mr. Sawers, grounded on his recusancy. These charges are before Government and the Commander-in-Chief, and we understand it is not found an easy matter to decide how 'they should be dealt with.'"—*From the Calcutta Newspapers.*

### MEDICAL BENEVOLENT FUND SOCIETY OF IRELAND.

We received the following paper merely in time to make an extract from it, and to cordially recommend the subject to the serious attention of our professional brethren in Ireland.—*Editors.*

"*Dublin, Thursday, May* 26, 1842.

"A meeting of 'medical gentlemen, favourable to the establishment of a Benevolent Fund Society,' having been called by requisition, the following members of the profession assembled in the Examination Hall of the College of Surgeons, at one o'clock, P.M., viz:—

"Sir Henry Marsh, bart., Mr. Carmichael, Mr. Wilmot, Dr. Graves, Dr. O'Beirne, Dr. Jacob, Dr. J. Jacob, Maryborough; Mr. Collis, Dr. Shekleton, Dr. Kingsley, Roscrea; Dr. Ferguson, Mullingar; Dr. Walsh, Naas; Dr. Blackley, Armagh; Dr. Corbett, Innishannon; Dr. Sherwood, county Wicklow; Dr. O'Grady, Dr. Duncan, sen., Dr. Duncan, jun., Dr. Benson, Dr. Harrison, Dr. Mollan, Dr. Hargrave, Dr. Brady, Dr. Macdonnell, Dr. Clinton, Dr. Harvey, Mr. Williams, Dr. Stuart, Dr. Fitzpatrick, Dr. Stephens, Mr. M'Entire, &c., &c.

"Sir Henry Marsh was moved to the chair, and Dr. Benson was requested to act as secretary.

"The first resolution was moved by Dr. Kingsley, and seconded by Dr. Graves—

"Resolved—That the formation of a Medical Benevolent Fund Society, similar to that which is connected with the Provincial Medical Association of England, is loudly called for by the casualities to which our profession is exposed.

"Dr. Kingsley said, Mr. Chairman and gentlemen, I don't think I could advocate the first resolution, which I have the honour of proposing, better, than by drawing the attention of this highly respectable meeting to the annual report for 1840 of the Society for the Relief of the Widows and Orphans of Medical Men in London and its vicinity. This charitable institution has been in active operation for more than fifty years, and was established by the benevolent exertions of but seven members of the medical profession. Commencing with a very limited number of subscribers, and consequently with a very slender income, it has gradually assumed such an importance, and attained to such a degree of prosperity, as place it upon a level with some of the most influential charities of the metropolis; the funded property of the society amounting now to nearly forty-five thousand pounds, enables the directors to distribute, with the aid of annual subscriptions, above fifteen hundred pounds per annum, among thirty-one widows, fifteen orphans of deceased members, and one aged and distressed member. The degree of relief afforded by the society to its pensioners has, of course, varied with its means; the present allowance is £35 per annum to a widow, provided her income from other sources does not exceed £50 a year; to each of her children under fourteen years of age, £12 yearly is allowed; and, under some circumstances, an apprentice fee is usually granted upon application. The sum of £26,066 5s. has been distributed among persons eligible to receive assistance. In 1810 it was found that, reckoning from the first establishment of the society, 97 of its members had died, and that the applications for relief from widows and orphans amounted to 21, so that nearly *one-fourth* of those who had died, left their families destitute, and consequently dependent upon the funds of this charity. It is devoutly hoped that a knowledge of such facts as these may influence the wealthier members of the profession, in Ireland, without exception, to join their humbler brethren solely from motives of charity towards their less fortunate brethren, in establishing, on a permanent and firm basis—'*The Medical Benevolent Fund of Ireland.*' It is still more to be desired that members of the profession, just commencing their career, aware as they must be of the difficulties that beset their path, should contribute to the funds of a charitable institution, the advantages of which might, by *possibility*, be reflected back upon their own dearest connections, and more especially as the subscription is so small in amount, (one guinea annually,) as scarcely to be an object of consideration to any one in practice. This union of prudence and benevolence, in supporting a charitable institution, could hardly fail to afford comfort to themselves, and be approved of by their friends. These views of the utility of the society chiefly apply to the members of the medical profession, but to the wealthy and charitably disposed of all classes of the community, some considerations may be addressed in behalf of this charity, particularly as the real condition of the profession seems to be little, if at all understood by persons unconnected with it. Those who look only on the surface of society, and who hear of the large fortunes accumulated by a few successful medical practitioners, may think that the practice of the medical profession is at once the sure and, ready road to independence and wealth; but the appalling fact already stated that *one in four* of the members of the society for the relief of the widows and orphans of medical men, &c., has left a widow, or orphans, claimant upon its funds, sufficiently disproves this opinion. The public generally appear to have no knowledge of the difficulties, and consequently, no sympathy for the struggles of men, who, bred with the notions and feelings of gentlemen, educated at a great expence, and obliged at all hazards to keep up an appearance of respectability, are compelled to wait quietly and silently for that employment, which they either may not obtain at all, or too late in life to afford them an opportunity of providing for their families. When it is also considered that the medical man is almost under the necessity of entering into the married state early in life, and that he is also more than ordinarily exposed to the chances of disease and premature death, it will not be a matter of astonishment, that so many respectable members of the profession of all classes, have been found to have their families unprovided for, their last days embittered by disappointed hope, rendered doubly poignant by the necessity of concealing their wants from the eyes of a hard judging world. When these circumstances are duly considered, can it be doubted that those who have it in their power to relieve real distress, and who must, in their own persons, or in those most dear to them, have experienced the restoration to health, or mitigation of disease, which the zeal and assiduity of the medical profession has tended to effect—can it be doubted, that they will contribute their aid to the widows and orphans of the less fortunate practitioners, whose deaths are often caused solely by their unwearied endeavours to do good to their fellow-creatures? Neither must it be forgotten, that the widows who may apply for the relief, which I hope this society will soon be in a posture to afford, will be persons taken from the better educated classes of society, who would prefer enduring in silence the privation of their accustomed comforts, to urging their claims upon public charity by making known their distress. I will not trespass further on the time of the meeting, but will read the resolution I have the great pleasure of proposing."

---

On the Preparations of the Indian Hemp, or Gunjah, (Cannabis Indica), their Effects on the Animal System in Health and Disease, &c., &c. By W. B. O'Shaughnessy, M.D. 8vo. sewed, pp. 38, 1843.

The narcotic effects of hemp are extensively known in South Africa, America, Turkey, Egypt, Asia, India, Malay, &c. But in Western Europe its use and abuse are little known. The extraordinary symptoms produced by the Indian hemp, or bang, depend on a resinous secretion with which it abounds, and this is totally absent in the European hemp. This difference appears to our author to depend on climate. The history and botanical characters of this medical substance are ably traced by the author, so is an account of its popular or intoxicating abuses. The bang is prepared by mixing powder of hemp with pepper, cucumber, melon seeds, sugar, milk, and water, when it is fit to make a man a beast.

" From this beverage intoxication will ensue in half an hour. Almost invariably the inebriation is of the most cheerful kind, causing the person to sing and dance, to eat food with great relish, and to seek aphrodisiac enjoyments. In persons of a quarrelsome disposition it occasions, as might be expected, an exasperation of their natural tendency. The intoxication lasts about three hours, when sleep supervenes. No nausea or sickness of the stomach succeeds, nor are the bowels at all affected; next day there is slight giddiness and much vascularity of the eyes, but no other symptom worth recording."

The natives of India have several preparations or forms of hemp, some of which they smoke instead of drinking.

Dr. O'S. made several experiments on animals, which shewed that while carnivora and fish, invariably exhibited the intoxicating influences of the drug, graminivora, as the horse, deer, monkey, goat, sheep, cow, &c., experienced only trivial effects, even from the largest doses. These experiments encouraged him to try its effects on man. We shall glance at some of the cases treated, chiefly in the Clinical Hospital of the Medical College, Calcutta.

*Cases of Rheumatism.*—Two cases of acute, and one of the chronic form, were treated. In the two former, little relief had been obtained by the common modes of treatment—depletion, Dover's powder, antimonials, &c. The same with the chronic case. One grain of the resin of hemp, in solution, was given to each of the three patients. In two hours, one of them was reported as becoming very talkative—singing songs—calling out for extra supplies of food, &c. The other two remained unaffected, in four hours the first patient was falling asleep. In six hours he was found quite insensible, but breathing regularly. Of the other two patients, one was asleep, the other free from any symptoms of intoxication. On raising up the arm of the first patient, Dr. O'S. found it cataleptic—in short, the whole body was in this curious state. This condition obtained for a few hours, when consciousness and voluntary motion returned, and the patient was soon well. The third man, who resisted the effects of the hemp resin, was found to be a gunjah or hemp smoker by habit. They all recovered, without any bad consequences. An old muscular cooley, a rheumatic malingerer, was next experimented on, by the exhibition of half a grain of the hemp-resin. In two hours he became talkative and musical—sang songs—told stories—ate double allowance of dinner, and sought other indulgences not on the catalogue of hospital fare. All ended in sound sleep, and he was discharged next morning.

*Hydrophobia.*—A terrible case of hydrophobia was treated by the resin in question, and, after five days exhibition of largish doses—sometimes two grains every hour or two, the hydrophobic symptoms were overcome; but the unfortunate patient, (a native doctor himself), fell a victim to coma. As far as can be judged from this and some other cases of the kind, we are now likely to possess a *Euthanasia*, if not a remedy, for the most torturing as well as fatal affliction to which humanity is subject.

Some of the pupils at the hospital now began to experiment on themselves and others, with the hemp-resin. The results in some cases were highly ludicrous. We shall quote a sample.

" In one pupil, Dinonath Dhur, a retiring lad of excellent habits, ten drops of the tincture, equal to a quarter of a grain of the resin, induced, in twenty minutes, the most amusing effects I ever witnessed. A shout of laughter ushered in the symptoms, and a transitory state of cataleptic rigidity occurred for two or three minutes. Summoned to witness the effects, we found him enacting the part of a Rajah giving orders to his courtiers; he could recognise none of his fellow students or acquaintances; all to his mind seemed as altered as his own condition: he spoke of many years having passed since his student's days: described his teachers and friends with a piquancy which a dramatist would envy; detailed the adventures of an imaginary series of years, his travels, his attainment of wealth and power; he entered on discussions on religious, scientific, and political topics, with astonishing eloquence, and disclosed an extent of knowledge, reading, and a ready apposite wit, which those who knew him best were altogether unprepared for. For three hours and upwards he maintained the character he at first assumed, and with a degree of ease and dignity perfectly becoming his high situation. A scene more interesting it would be difficult to imagine. It terminated nearly as suddenly as it commenced, and no headache, sickness, or other unpleasant symptom followed the innocent excess."

The remedy was tried in cholera, by several practitioners, and with considerable success.

*Traumatic Tetanus.*—It was in this dire and generally incurable malady, that the hemp-resin and tincture evinced the most salubrious effects. Many cases are detailed, and a great majority were saved. In this class of diseases, the doses were raised to two or three grains, every two or three hours. The remedy was tried, with more or less effect, in some cases of infantile convulsions, delirium tremens, &c., for which we must refer to the paper in question. A sufficient supply of the extract and tincture, prepared from the fresh hemp-tops, may be had at Mr. Squire's, in Oxford-street, who will enable the medical officers of public institutions to give a fair clinical trial to this remarkable and potent, though, it appears, safe medicinal agent. We fear that the Indian hemp will, from some cause or other, prove less effective in this than in its native climate; but the facts brought forward, with so much industry and candour, by Dr. O'Shaughnessy, are deserving of immediate attention and extensive trial, especially by hospital physicians and surgeons.

---

### Mesmerism; its Pretensions and Effects, &c. By John Brown, M.D. of Boston, Lincolnshire. 1843.

The Mesmero-mania has nearly dwindled, in the metropolis, into anile fatuity; but lingers in some of the provinces, with the *gobe-mouches* and chaw-bacons, who, after gulping down a pound of fat pork, would, with well greased gullets, swallow such a lot of mesmeric mummery as would choke an alligator or a boa constrictor. A worthy disciple, and operator on a narrow scale, (Mr. Small,) has been exhibiting, in his shop at Boston, to a gaping multitude, and with a merry-andrew, whom he has had in *training* for some time. The following passage from Dr. Brown's brochure contains enough of this mesmeric farce:—

"A gentleman at one of Messrs. Small and Sharp's public exhibitions said, 'Why do you always exhibit the same person? take one of us;' when Mr. Small replied, 'would it be reasonable to expect as much from an untrained horse, as a trained one?' Thank you, Mr. Small, the cat is out of the bag; *training* is the word which expresses all the wonders of Mesmerism.

"Part of the exhibition now consists in Mr. Small sitting down to parody his own miracles: he shuts his eyes, holds out his hand with a glass in it, and has a copper cap fired off, without flinching or starting. 'There,' says he, 'I can do all these, and I should think John an impostor myself, if he did no more; but

I shall now exhibit to you an experiment, which you must all, I am sure, deem decisive ;' John is now made to stand up in his sleep, blindfolded, a small circle is cleared around, Mr. Small takes off his shoes, puts himself in attitude, waves his hands about, walks backward round and round, and John follows : this decisive experiment has been daily exhibited, to the wonder of visitors who cannot make it out, and believe it to be magnetic attraction, but every one ought to see that really there is no attraction in it, for as the two performers approach near to each other, the attraction, instead of increasing, ceases; when Mr. Small stands still, John stands still, because John knows very well that the next step would incommode Mr. Small: John is a well trained performer—an accomplished performer, and well deserves the shillings he picks up at private exhibitions. On John being asked if he could follow Mr. Small on saw-dust, he said 'he could follow Mr. Small very well on a boarded floor, but neither he nor any one else could follow him on saw-dust.'"

